# The risk of malignancy and its incidence in early rheumatoid arthritis patients treated with biologic DMARDs

**DOI:** 10.1186/s13075-017-1482-y

**Published:** 2017-12-15

**Authors:** Soo-Kyung Cho, Jiyoung Lee, Minkyung Han, Sang-Cheol Bae, Yoon-Kyoung Sung

**Affiliations:** 1 Hanyang University Hospital for Rheumatic Diseases, 222-1 wangsimni-ro, Seongdong-gu, Seoul, 04763 South Korea; 2 Clinical Research Center for Rheumatoid Arthritis (CRCRA), 222 wangsimni-ro, Seongdong-gu, Seoul, 04763 South Korea

**Keywords:** Rheumatoid arthritis, Malignancies, Disease-modifying anti-rheumatic drugs, Biologic DMARDs

## Abstract

**Background:**

Treatment with disease-modifying anti-rheumatic drugs (DMARDs) has raised concerns about the risk of malignancies in rheumatoid arthritis (RA) patients. However, the association between biologic DMARDs (bDMARDs) and malignancy in previous reports remains controversial. Therefore we aimed to estimate the incidence of malignancy in early RA patients and to evaluate the relative risk of malignancy with use of bDMARDs.

**Methods:**

A retrospective cohort of incident RA patients was established using the Korean National Claims Database. Among a total of 14,081 RA patients identified, 1684 patients with a history of malignancy were excluded. We calculated the incidence rate of overall and individual malignancies. The standardized incidence ratio (SIR) of malignancies in bDMARD users was compared to that in nonusers. Multivariable logistic regression analysis was used to evaluate the impact of bDMARDs on the development of malignancies in early RA patients.

**Results:**

A total of 12,397 early RA patients without a history of malignancy were enrolled. During 41,599 person-years (PY) of follow-up, 725 malignancies developed in 561 patients (174.3/10,000 PY) and 21 hematologic malignancies developed (5.0/10,000 PY). Patients treated with bDMARDs had a significantly lower incidence of overall malignancy compared to those not treated with bDMARDs (SIR 0.45 (95% CI 0.28–0.70)). However, this relationship was not significant with regard to hematologic malignancies (SIR 2.65 (95% CI 0.55–7.76)). On multivariable analysis, bDMARD use was a protective factor against the development of overall malignancy (odds ratio 0.42 (95% CI 0.25–0.73)). However, bDMARD use had no significant protective effect against the development of hematologic malignancies (odds ratio 1.69 (95% CI 0.38–7.59)).

**Conclusions:**

In early RA patients, bDMARD use decreases the overall risk of developing malignancies; however, it does not affect the risk of developing hematologic malignancies.

**Electronic supplementary material:**

The online version of this article (doi:10.1186/s13075-017-1482-y) contains supplementary material, which is available to authorized users.

## Background

Patients with rheumatoid arthritis (RA) are at increased risk of developing certain malignancies, including lymphoma, leukemia, and lung cancer [1–3]. This predisposition for malignancy is attributed to the immune dysregulation and/or chronic inflammation in RA, which increases cell proliferation, mutagenesis, oncogene activation, and angiogenesis [4]. Moreover, the long-term use of disease-modifying anti-rheumatic drugs (DMARDs), including methotrexate (MTX) and tacrolimus, is thought to increase risk of hematologic malignancies [5, 6]. Recently, biologic DMARDs (bDMARDs) have become effective treatment options for RA patients with an inadequate response to MTX [7–9]. However, there are also concerns regarding malignancy with the use of these biologic agents given their target-specific inhibition of the immune system. Among various bDMARDs, concern is greatest with anti-tumor necrosis factor (anti-TNF) therapies, given the established role of TNF in tumor progression and surveillance [10].

A recent meta-analysis of randomized trials found that treatment with TNF inhibitors was associated with a 2–3.3-fold elevated risk of malignancy compared to non-TNF inhibitor therapies [11, 12]. However, other meta-analyses and observational studies have not identified a substantial increased risk of overall malignancies with TNF inhibitor use [13–15]. There are few studies that address cancer risk with the use of other (non-TNF inhibitor) bDMARDs. The only such studies that have been published are relatively short-term randomized trials that address the cancer risks of abatacept [16], tocilizumab [17], and rituximab [18] in RA. Also, recent postmarketing surveillance found that treatment with tocilizumab did not increase the risk of malignancy in RA patients [19].

Observational registry-based data also suggest that TNF inhibitors do not increase the risk of malignancy in patients with RA [20]. Furthermore, recent reports have suggested that TNF inhibitors actually decrease the risk of malignancies, with the exception of hematologic malignancies [21, 22]. Therefore, the association between bDMARDs and malignancy remains controversial [23]. It is also difficult to assess the influence of bDMARDs on malignancy development in patients with established RA because of the potential confounding influences of conventional DMARDs, long-term disease duration, or individual biases such as malignancy history.

Therefore, we aimed to determine the incidence of malignancies in newly diagnosed RA patients, and to evaluate the impact of bDMARDs on malignancy development.

## Methods

### Data source

An inception cohort of early RA patients was built based on the Health Insurance Review and Assessment (HIRA) database of South Korea. All people in South Korea are eligible for coverage under the National Health Insurance Program. Therefore, a total number of 47 million people, or over 96.3% of the total population, were included in the HIRA database [24]. This database contains individual beneficiary information, as well as healthcare service information including diagnosis, procedures, prescriptions, and tests. The HIRA Research Ethics Committee of South Korea approved this study protocol (NSCR-2014-3).

### Study population and outcomes

#### Study population

**Fig. 1:**
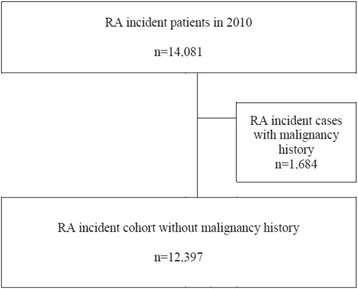
Patient selection flow. RA rheumatoid arthritis

Patients with RA were identified using the diagnostic code for RA and records of prescriptions for any DMARDs. This RA definition was established according to our validated operational definition of RA in the HIRA database [25]. An inception cohort of incident RA patients was constructed containing patients who were identified as RA patients in 2010, were disease-free for 1 year prior to the index date, and received continuous treatment for > 3 years from the index date onward. The index date was defined by the initiation of the first DMARD in 2010. Patients were observed until the development of a malignancy or until the end of the study period (in December 2013), whichever came first. A total of 14,081 early RA patients were identified. Patients with a history of malignancy (*n* = 1684) for at least 12 months prior to the index date were excluded (Fig. 1).

#### Definition of malignancy

The development of malignancy was defined as the appearance of the respective ICD-10 code between the index date and the last date of observation [26] (Additional file 1: Table S1). Malignancies were divided according to the involved systemic organs.

### Study design

#### Retrospective cohort study

The incidence rate (IR) of malignancy was calculated for early RA patients without a history of malignancy in 2010. As described previously, patients were followed until the development of malignancy or until the last observational date. Therefore, patients without malignancy development were followed for 3–4 years. Patients were divided into two groups according to bDMARD exposure during the observational period: bDMARD ever-users and bDMARD nonusers. Patients with any use of bDMARDs during the observational period were included in the bDMARD ever-user group, while those without bDMARD use were placed in the bDMARD nonuser group. The IR of malignancies was calculated in each group. The standardized incidence ratio (SIR) of bDMARD users was calculated based on bDMARD nonusers. Nearly 95% of patients were bDMARD nonusers; therefore, the nonusers were considered the standard RA population.

#### Nested case–control study

A nested case–control study was performed on this inception cohort for early RA patients in order to evaluate the impact of bDMARDs on the development of malignancies. Patients were divided into two groups (patients with and without malignancies) according to the development of malignancies during the observation period of > 3 years. Univariable and multivariable analyses were performed to identify the impact of bDMARD use on the development of all malignancies, and hematological malignancies specifically.

### Statistical analysis

The crude IR of each malignancy (per 10,000 PY), and the respective 95% confidence intervals (CIs) were calculated. First, we compared the demographic and clinical characteristics between the bDMARD ever-user and nonuser groups using the chi-square test and the independent *t* test. In order to estimate the relative risk of developing malignancies in bDMARD users compared to nonusers, we calculated the SIR (the number of observed malignancies per number of expected malignancies) after adjusting for age and sex. The 95% CI was determined assuming that the frequency of observed cases followed a Poisson distribution.

In the case–control study, multivariable logistic regression models and Poisson regression models were used to identify the impact of bDMARD use on the development of overall and hematological malignancies. These models were performed after adjusting for the following parameters at baseline: age, sex, healthcare utilization (such as insurance type), type of institution, department type, number of comorbidities, and medication (including MTX, corticosteroids, and NSAIDs).

All analyses were performed using SAS 9.2 (SAS Institute, Cary, NC, USA). *p* < 0.05 was considered statistically significant.

## Results

### Baseline characteristics of the study population

A total of 12,397 patients with early RA (41,599 PY) were included in analysis. The baseline characteristics of the study population are summarized in Table 1. The mean patient age (standard deviation (SD)) was 52.7 (13.6) years and 76.8% of the patients were female. Hydroxychloroquine was the most frequently used DMARD (56.6%), followed by methotrexate (MTX) at 42.6% and sulfasalazine at 21.8%. In addition, 71.4% of the patients used corticosteroids and 91.7% used NSAIDs at baseline.

**Table 1:** Baseline characteristics of early rheumatoid arthritis patients

Variable	Total (*N* = 12,397)	bDMARD ever-user (*n* = 714)	bDMARD nonuser (*n* = 11,683)	*p*
Age (years)	52.7 ± 13.6	47.5 ± 15.1	53.0 ± 13.4	< 0.01
Female sex	9525 (76.8)	456 (63.9)	9069 (77.6)	< 0.01
Insurance type				< 0.01
Health insurance	11,661 (94.1)	682 (95.5)	10,979 (94.0)	
Medicaid	736 (5.9)	32 (4.8)	704 (6.0)	
Type of institution				< 0.01
Tertiary hospital	2646 (21.3)	231 (32.4)	2415 (20.7)	
General hospital	2430 (19.6)	161 (22.6)	2269 (19.4)	
Community hospitals/clinics/other	7321 (59.1)	322 (45.1)	6999 (59.9)	
Department				< 0.01
Internal medicine	5064 (40.9)	362 (50.7)	4702 (40.3)	
Orthopedic surgery	5839 (47.1)	265 (37.1)	5574 (47.7)	
Other	1494 (12.1)	87 (12.2)	1407 (12.0)	
Number of comorbidities	0.84 ± 1.01	0.80 ± 0.93	0.85 ± 1.01	0.17
0	5755 (46.4)	338 (47.3)	5417 (46.4)	
1	3993 (32.2)	231 (32.4)	3762 (32.2)	0.76
≥ 2	2649 (21.4)	145 (20.3)	2504 (21.4)	
CCI score	2.0 ± 1.2	1.9 ± 1.1	2.0 ± 1.2	0.14
Methotrexate use	5283 (42.6)	437 (61.2)	4846 (41.5)	< 0.01
Methotrexate dose (mg/week)	9.8 ± 3.0	10.9 ± 3.1	9.7 ± 3.0	< 0.01
Dosage = 0	7114 (57.5)	277 (38.8)	6837 (58.6)	
0 < dosage < 10	2452 (19.8)	147 (20.6)	2305 (19.8)	< 0.01
Dosage ≥ 10	2814 (22.7)	290 (40.6)	2524 (21.6)	
Hydroxychloroquine	7016 (56.6)	339 (47.5)	6677 (57.2)	< 0.01
Sulfasalazine	2704 (21.8)	296 (41.5)	2408 (20.6)	< 0.01
Bucillamine	737 (5.9)	31 (4.3)	706 (6.0)	0.07
Leflunomide	687 (5.5)	84 (11.8)	603 (5.2)	< 0.01
Tacrolimus	87 (0.7)	13 (1.8)	74 (0.63)	< 0.01
Oral corticosteroid use	8851 (71.4)	549 (76.9)	8302 (71.1)	< 0.01
Corticosteroid dose (mg/day)	7.4 ± 4.8	7.8 ± 4.1	7.4 ± 4.8	0.04
Dosage = 0	3546 (33.3)	165 (26.0)	3381 (33.7)	
0 < dosage < 5	1244 (11.7)	39 (6.1)	1205 (12.0)	< 0.01
Dosage ≥ 5	5874 (55.1)	431 (67.9)	5443 (54.3)	
NSAID use	11,366 (91.7)	671 (94.0)	10,695 (91.5)	0.03

Data expressed as mean ± standard deviation or number (percentage)

*bDMARD* biologic disease-modifying anti-rheumatic drug, *CCI* Charlson comorbidity index, *NSAID* nonsteroidal anti-inflammatory drug

A total of 714 patients (5.8%) had used bDMARDs. Only TNF inhibitors were allowed for use as a first-line bDMARD agent through December 2013 in South Korea. Therefore, all of the bDMARD users in this study were assumed to have been exposed to a TNF inhibitor. The bDMARD ever-user group was younger (mean age 47.5 vs 53.0 years, *p* < 0.01) and had a higher proportion of men (36.1% vs 22.4%, *p* < 0.01) than did the bDMARD nonuser group. There was no significant difference between the ever-user and nonuser group with regard to comorbidities. However, drug utilization varied considerably between the two groups. Both the prevalence of MTX use (61.2% vs 41.5%, *p* < 0.01) and its dosages (10.9 ± 3.1 vs 9.7 ± 3.0, *p* < 0.01) were higher in bDMARD ever-users compared to those in bDMARD nonusers. Oral corticosteroids and NSAIDs were also more frequently used in the bDMARD user group than they were in the bDMARD nonuser group.

### Incidence of malignancy in early RA patients

#### Incidence of malignancy in early RA patients

A total of 725 malignancies in 561 patients (200 in males and 361 in females) were diagnosed during the observation period. Among these, 140 patients had more than one malignancy. The total IR of overall malignancies in early RA patients was 174.3/10,000 PY (95% CI 161.6–187.0): 280.4/10,000 PY in men (95% CI 246.7–314.1) and 142.9/10,000 PY (95% CI 129.9–156.0) in women.

The involved organ systems differed according to sex. In men (*n* = 2872, 9486 PY), prostate malignancies were most common at 49.5/10,000 PY (95% CI 35.4–63.7), followed by lung cancer (34.8/10,000 PY (95% CI 22.9–46.7)), stomach cancer (32.7/10,000 PY (95% CI 21.2–44.2)), and colorectal cancer (29.5/10,000 PY (95% CI 18.6–40.4)). In women (*n* = 9525, 32,113 PY), the IR of thyroid cancer was highest at 25.5/10,000 PY (95% CI 20.0–31.1), followed by colorectal cancer (17.4/10,000 PY (95% CI 12.9–22.0)), breast cancer (11.8/10,000 PY (95% CI 8.1–15.6)), and pancreatic cancer (11.5/10,000 PY (95% CI 7.8–15.2)) (Table 2).

**Table 2:** Incidence rates of malignancy in early rheumatoid arthritis patients by sex

Type of malignancy	Total (*N* = 12,397)		Male (*n* = 2872)		Female (*n* = 9525)	
	*N*	IR^a^ (95% CI)	*N*	IR^a^ (95% CI)	*N*	IR^a^ (95% CI)
All malignancies	725	174.3 (161.6–187.0)	266	280.4 (246.7–314.1)	459	142.9 (129.9–156.0)
Lip, oral cavity, and pharynx	9	2.2 (0.8–3.6)	5	5.3 (0.7–9.9)	4	1.2 (0.0–2.5)
Esophagus	3	0.7 (0.1–1.5)	3	3.2 (0.4–6.7)	–	–
Stomach	61	14.7 (11.0–18.3)	31	32.7 (21.2–44.2)	30	9.3 (6.0–12.7)
Colon and rectum	84	20.2 (15.9–24.5)	28	29.5 (18.6–40.4)	56	17.4 (12.9–22.0)
Liver	52	12.5 (9.1–15.9)	22	23.2 (13.5–32.9)	30	9.3 (6.0–12.7)
Gallbladder	10	2.4 (0.9–3.9)	2	2.1 (0.8–5.0)	8	2.5 (0.8–4.2)
Pancreas	50	12.0 (8.7–15.4)	13	13.7 (6.3–21.2)	37	11.5(7.8–15.2)
Larynx	5	1.2 (0.1–2.3)	5	5.3 (0.7–9.9)	–	–
Lung	60	14.4 (10.8–18.1)	33	34.8 (22.9–46.7)	27	8.4 (5.2–11.6)
Breast	39	9.4 (6.4–12.3)	1	1.1 (1.0–3.1)	38	11.8 (8.1–15.6)
Cervix uteri	17	4.1 (2.1–6.0)	0	–	17	5.3 (2.8–7.8)
Corpus uteri	4	1.0 (0.0–1.9)	0	–	4	1.2 (0.0–2.5)
Ovary	13	3.1 (1.4–4.8)	0	–	13	4.0 (1.8–6.2)
Prostate	47	11.3 (8.1–14.5)	47	49.5 (35.4–63.7)	0	–
Testis	3	0.7 (0.09–1.5)	3	3.2 (0.4–6.7)	0	–
Kidney	8	1.9 (0.6–3.3)	4	4.2 (0.1–8.3)	4	1.2 (0.0–2.5)
Bladder	14	3.4 (1.6–5.1)	8	8.4 (2.6–14.3)	6	1.9 (0.4–3.4)
Brain and CNS	5	1.2 (0.1–2.3)	1	1.1 (1.0–3.1)	4	1.2 (0.0–2.5)
Thyroid	87	20.9 (16.5–25.3)	5	5.3 (0.7–9.9)	82	25.5 (20.0–31.1)
Hematologic malignancy	21	5 (2.9–7.2)	6	6.3 (1.3–11.4)	15	4.7 (2.3–7.0)
Hodgkin lymphoma	1	0.2 (0.2–0.7)	0	–	1	0.3 (0.3–0.9)
Non-Hodgkin lymphoma	13	3.1 (1.4–4.8)	4	4.2 (0.1–8.3)	9	2.8 (1.0–4.6)
Multiple myeloma	1	0.2 (0.2–0.7)	0	–	1	0.3 (0.3–0.9)
Leukemia	6	1.4 (0.3–2.6)	2	2.1 (0.8–5.03)	4	1.2 (0.0–2.47)
Others	133	32.0 (26.5–37.4)	49	51.7 (37.2–66.1)	84	26.2 (20.6–31.8)

*IR* incidence rate, *CI* confidence interval, *CNS* central nervous system

^a^Incidence rate represents the incident cases per 10,000 person-years

#### Incidence rate of malignancy in bDMARD users

The IRs of malignancy according to the use of bDMARDs are presented in Table 3. In bDMARD users (*n* = 714, 2454 PY), the IR of overall malignancy was 81.5/10,000 PY (95% CI 45.8–117.2). In contrast, the IR of overall malignancy in nonusers was 180.1/10,000 PY (95% CI 166.8–193.4). The SIR of overall malignancy in bDMARD users was only 0.5 (95% CI 0.3–0.7). However, for hematologic malignancy, the SIR in bDMARD users was 2.9 (95% CI 0.4–10.5) for lymphoma and 3.2 (95% CI 0.01–18.0) for leukemia, although these were not significant.

**Table 3:** Incidence rate of malignancies in early RA patients according to bDMARD use

Type of malignancy	bDMARD ever-user (*n* = 714)		bDMARD nonuser (*n* = 11,683)		Standardized incidence rate
	*N*	IR^a^ (95% CI)	*N*	IR^a^ (95% CI)	SIR (95% CI)^b^
All malignancies	20	81.5 (45.8–117.2)	705	180.1 (166.8–193.4)	0.5 (0.3–0.7)
Lip, oral cavity, and pharynx	0	–	9	2.3 (0.8–3.8)	–
Esophagus	0	–	3	0.8 –(0.1–1.6)	–
Stomach	3	12.2 (1.6–26.0)	58	14.8 (11.0–18.6)	0.8 (0.2–2.4)
Colon and rectum	1	4.07 (3.9–12.0)	83	21.2 (16.6–25.8)	0.2 (0.0–1.1)
Liver	1	4.07 (3.9–12.0)	51	13.0 (9.5–16.6)	0.3 (0.01–1.7)
Gallbladder	–	–	10	2.6 (1.0–4.1)	–
Pancreas	1	4.07 (3.9–12.0)	49	12.5 (9.0–16.0)	0.3 (0.01–1.8)
Larynx	0	–	5	1.3 (0.2–2.4)	–
Lung	3	12.2 (1.6–26.0)	57	14.6 (10.8–18.3)	0.8 (0.2–2.5)
Breast	3	12.2 (1.6–26.0)	36	9.2 (6.2–12.2)	1.3 (0.3–3.9)
Cervix uteri	0	–	17	4.3 (2.3–6.4)	–
Corpus uteri	0	–	4	1.0 (0.0–2.0)	–
Ovary	0	–	13	3.3 (1.5–5.1)	–
Prostate	0	–	47	12.0 (8.6–15.4)	–
Testis	0	–	3	0.8 (0.1–1.6)	–
Kidney	0	–	8	2.0 (0.6–3.5)	–
Bladder	0	–	14	3.6 (1.7–5.5)	–
Brain and CNS	0	–	5	1.3 (0.2–2.4)	–
Thyroid	1	4.1 (3.9–12.0)	86	22.0 (17.3–26.6)	0.2 (0.0–1.03)
Hematologic malignancy	3	12.2 (1.6–26.0)	18	4.6 (2.5–6.7)	2.7 (0.6–7.8)
Hodgkin lymphoma	0	–	1	0.3 (0.2–0.8)	–
Non-Hodgkin lymphoma	2	8.2 (3.1–19.4)	11	2.8 (1.1–4.5)	2.9 (0.4–10.5)
Multiple myeloma	0	–	1	0.3 (0.2–0.8)	–
Leukemia	1	4.1 (3.9–12.0)	5	1.3 (0.2–2.4)	3.2 (0.1–18.0)
Others	4	16.3 (0.3–32.3)	129	33.0 (27.3–38.6)	0.5 (0.1–1.3)

*bDMARD* biologic disease-modifying anti-rheumatic drug, *RA* rheumatoid arthritis, *IR* incidence rate, *SIR* standardized incidence rate, *CI* confidence interval, *CNS* central nervous system

^a^Incidence rates represent incident cases per 10,000 person-years

^b^Standardized incidence rate was compared to bDMARD nonuser

#### Impact of malignancy on the development from bDMARD use

The baseline characteristics of early RA patients according to the development of malignancies are summarized in Additional file 2: Table S2. In multivariable analysis adjusted for various factors, bDMARD use was associated with a low risk of malignancy (odds ratio (OR) 0.42 (95% CI 0.25–0.73)), while older age (OR 1.04 (95% CI 1.03–1.04)) and male (OR 1.88 (95% CI 1.57–2.25)) increased the risk of malignancy development (Table 4). However, bDMARD use was not protective in the development of hematologic malignancies (OR 1.69 (95% CI 0.38–7.59)) (Table 4).

**Table 4:** Risk factors for the development of malignancy in early RA patients^a^

Variable	Malignancies		Hematologic malignancies	
	Crude	Adjusted	Crude	Adjusted
	OR (95% CI)	OR (95% CI)	OR (95% CI)	OR (95% CI)
Age	1.04 (1.03–1.05)	1.04 (1.03–1.04)	1.02 (0.99–1.06)	1.02 (0.98–1.05)
Male sex	1.90 (1.59–2.27)	1.88 (1.57–2.25)	1.55 (0.59–4.07)	1.41 (0.53–3.74)
Comorbidities	1.66 (1.39–1.99)	1.34 (1.11–1.61)	2.42 (0.87–6.73)	2.29 (0.80–6.52)
Medications				
bDMARD ever use	0.41 (0.24–0.70)	0.42 (0.25–0.73)	1.91 (0.44–8.27)	1.69 (0.38–7.59)
Methotrexate use	0.90 (0.76–1.07)	0.93 (0.78–1.12)	1.21 (0.49–2.97)	1.06 (0.41–2.74)
Corticosteroid use	1.03 (0.86–1.25)	1.05 (0.86–1.28)	1.12 (0.40–3.11)	1.02 (0.35–2.95)
NSAID use	1.22 (0.88–1.71)	1.16 (0.83–1.63)	1.61 (0.22–12.09)	2.00 (0.26–15.48)

*RA* rheumatoid arthritis, *OR* odds ratio, *CI* confidence interval, *bDMARD* biologic disease-modifying anti-rheumatic drug, *NSAID* nonsteroidal anti-inflammatory drugs

^a^Adjusted by type of healthcare utilization including insurance, type of institution, type of department

## Discussion

In this large population study, the IR of all malignancies in early RA patients was 174.3/10,000 PY, while that of hematologic malignancies was 5.0/10,000 PY. The risk of malignancy in bDMARD users was lower than that of nonusers, with the exception of hematologic malignancies.

The risk of malignancy in RA patients appears to vary widely among ethnicities and geographical regions with respect to genetic predisposition and environmental factors [27, 28]. The IR of overall malignancy in this study is higher than that obtained in American studies using a national database (129.6/10,000 PY) [29]. In particular, the IR of overall malignancy in this study was much higher than that in Japan (67.5–82.0/10,000 PY) [6, 30] using registries. These differences are likely influenced by the different data sources. We used the national claims database, which does not have any loss to follow-up with regard to malignancy. Compared to the national database, a higher rate of loss to follow-up is the main cause of underestimation of severe outcomes in the disease-specific cohort study [31, 32]. One recent comparison study of five RA registries from several different countries based on considering different study designs found that the IRs for all malignancies, excluding skin cancers, were highly consistent across studies [33]. Use of the National Cancer Screening System is another important factor in the increased incidence of malignancies in our study compared to those in other studies. Interestingly, thyroid cancer was the most common type of malignancy in Korean patients with RA. This finding is quite different from that identified in other countries [2, 30, 34]. This trend for a high incidence of thyroid cancer has been seen in the general population in Korea [35]. South Korea has an aggressive screening system for the early detection of malignancies, including thyroid cancer. This screening program may contribute to the increased incidence of thyroid cancer detected in this study [36].

The influence of bDMARDs on cancer development has been unclear [11–18, 20]. Several recent reports have suggested that there is a lower IR of malignancy in TNF inhibitor users compared to that in bDMARD nonusers, or in the general population. The IR per 10,000 PY of malignancies was lower in TNF inhibitor users (81/10,000 PY) compared with bDMARD nonusers (117/10,000 PY) in the BSRBR [20], and was 53.5/10,000 PY in bDMARD users and 74.1/10,000 PY in bDMARD nonusers from the Taiwan national database [22]. The Japanese cohort for RA patients given bDMARDs (SECURE) recently also reported that the risk of overall malignancies was significantly lower in bDMARD users than it was in the general population [30]. Therefore, our finding of a beneficial effect of bDMARDs with regard to cancer development is consistent with these recent reports.

In our study, patients treated with bDMARDs were also more frequently exposed to MTX, corticosteroids, and NSAIDs (at higher dosages) than were nonusers of bDMARDs (Additional file 2: Table S2). Therefore, bDMARD users appear to have higher RA disease activity than do nonusers based on these medication requirements. Inflammation is believed to play a key role in the development of malignancy [4]. Treatment with bDMARDs, however, can potentially reduce this risk through appropriate activity control, reducing the need for other treatments in the long term.

However, our results also demonstrate that the risk of hematologic malignancies was unaffected by bDMARD use. In fact, the number of hematologic malignancies tended to be higher in bDMARD users compared to nonusers, although this was not statistically significant. Our study was limited to early RA patients with 3–4 years of follow-up. Therefore, the long-term effect of bDMARDs on the development of hematologic malignancies cannot be confirmed with this study. Further long-term observational studies are needed to clarify the risk of bDMARD use on the development of hematologic malignancies.

There is a well-recognized association between malignancy and RA itself. In this study, we included patients who were early in their RA disease course in order to control the disease duration and to avoid the influences of long-term exposure of conventional DMARDs on the incidence of malignancy. Our data did not show a meaningful difference in the IR of all malignancies in patients with early RA compared to that of established RA patients (reported in several registries). According to previous studies, early RA patients (SRR and NOAR) and established RA patients (CORRONA and IORRA) have similar incidences of malignancy [33].

Our study has several limitations. First, we did not adjust for the time of bDMARD use as a time-dependent variable. We decided not to use drug duration as a time-dependent variable because of the relatively short length of our observational period. There has not been any evidence for a relationship between bDMARD exposure time and the risk of malignancy development based on many prior studies. Moreover, since our study showed that ever-exposure to bDMARDs was not a risk factor but a protective factor for the development of overall malignancies, we did not analyze bDMARD exposure as a time-dependent variable. Regardless, a longer study is needed to determine the long-term effects of bDMARD use in RA patients. A second limitation of this study is that we did not perform subgroup analysis according to the type of bDMARD. Some types of bDMARDs are noted to be risk factors for certain malignancies. However, we were unable to separate patients by specific bDMARD agents, because most of these patients were treated with TNF inhibitors, which are first-line bDMARDs according to our reimbursement guideline. Therefore, there may be few patients exposed to other bDMARDs in this study period. In addition, we could not adjust the clinical information, such as disease activity, because of the limitation of the claims database. However, we adjusted for the use of specific medications as surrogate markers of disease activity. We also adjusted comorbidities more accurately than was done in previous studies, in which adjustment was based on patients’ reports or on the medical records of a single hospital. Finally, we could not compare the IR in the overall RA patients with that in the general population. A study matching population characteristics or observational periods will be needed for that.

This study also has several strengths. First, our study population was limited to patients who were early in the course of RA. Therefore, we could control for confounders such as disease duration and exposure time of various conventional DMARDs. Second, by excluding patients with a history of malignancy, we reduced the indication bias for bDMARD use. This strategy was meaningful when comparing the risks of malignancy development based on bDMARD use. A third strength of this study is that we used the National Claims Database, which covers the entire Korean population and therefore avoids selection bias. This large sample size and the availability of long-term records enhanced the statistical power to look at rare events and the accuracy of our study. In addition, this database prevents the underestimation of malignancy incidence and minimizes loss to follow up, while there is a risk of under-reporting of malignancies if patients leave the registry or are not adequately followed up in registry observational data. Furthermore, our data provide additional evidence leading to robust conclusions regarding the risk of malignancy in patients treated with bDMARDs because the pattern of concomitant DMARDs varies according to countries.

## Conclusion

The use of bDMARDs in early RA patients is associated with reduced risk of malignancy development, with the exception of hematological malignancies.

## Additional files

Additional file 1: Table S1. Presenting diagnostic codes for malignancies. (DOCX 36 kb)

Additional file 2: Table S2. Presenting comparison of baseline characteristics between patients diagnosed with malignancies and patients without malignancies. (DOCX 19 kb)

## Abbreviations

RA: Rheumatoid arthritis
bDMARD: Biologic disease-modifying anti-rheumatic drug
SIR: Standardized incidence ratio
PY: Person-years
DMARD: Disease-modifying anti-rheumatic drug
MTX: Methotrexate
TNF: Tumor necrosis factor
HIRA: Health Insurance Review and Assessment
IR: Incidence rate
NSAID: Nonsteroidal anti-inflammatory drug
OR: Odds ratio
