# Supplementary material for: The risk of malignancy and its incidence in early rheumatoid arthritis patients treated with biologic DMARDs
Source: Arthritis Res Ther. 2017 Dec 15;19:277. doi: 10.1186/s13075-017-1482-y (PMC5732524; doi:10.1186/s13075-017-1482-y)
Supplement: Supplementary file 1 — Presenting diagnostic codes for malignancies. (DOCX 36 kb) [file 13075_2017_1482_MOESM1_ESM.docx]

Additional file 1: Table S1. Diagnostic codes for malignancies

| Type of malignancy | ICD 10 code |
| --- | --- |
| all cancer | C00-C97 |
| lip, oral cavity and pharynx | C00-C14 |
| esophagus | C15 |
| stomach | C16 |
| colon and rectum | C18-C20 |
| liver | C22 |
| gallbladder | C23, C24 |
| pancreas | C25 |
| larynx | C32 |
| lung | C33, C34 |
| breast | C50 |
| cervix uteri | C53 |
| corpus uteri | C54 |
| ovary | C56 |
| prostate | C61 |
| testis | C62 |
| kidney | C64 |
| bladder | C67 |
| brain and CNS | C70-C72 |
| thyroid | C73 |
| Hodgkin lymphoma | C81 |
| non-Hodgkin lymphoma | C82-C85 |
| multiple myeloma | C90 |
| leukemia | C91-C95 |
| malignant neoplasms of lymphoid, hematopoietic and related tissue | C96 |
| Other | C17, C21, C26~C31, C35~C49, C51, C52, C55, C57~C60, C63, C65, C66, C68, C69, C74~80, C86~C89, C97 |

Additional file 2: Table S2. Comparison of baseline characteristics between patients diagnosed as malignancies and patients without malignancies

| Variables | Patients with malignancies (n=561) | No malignancies  (n=11,836) | *p-value* |
| --- | --- | --- | --- |
| Age (years, mean±SD) | 59.16 ± 12.19 | 52.37 ± 13.58 | <0.001 |
| Gender (female) | 361 (64.4) | 9,164 (77.4) | <0.001 |
| Type of insurance |  |  | 0.56 |
| Health insurance | 524 (93.4) | 11,137 (94.1) |  |
| Medicaid | 37 (6.6) | 699 (5.9) |  |
| Type of institution |  |  | 0.8 |
| Tertiary hospitals | 114 (20.3) | 2,532 (21.4) |  |
| General hospitals | 114(20.3) | 2,316 (19.6) |  |
| Community hospitals/clinics/other | 333 (59.4) | 6,988 (59.0) |  |
| Type of department |  |  | 0.05 |
| Internal medicine | 229 (40.8) | 4,835 (40.9) |  |
| Orthopedics | 247 (44.0) | 5,592 (47.3) |  |
| Other | 85 (15.2) | 1,409 (11.9) |  |
| Number of comorbidities | 1.17 ± 1.17 | 0.83 ± 1.00 | <0.001 |
| 0 | 195 (34.8) | 5,560 (47.0) | <0.001 |
| 1 | 180 (32.1) | 3,813 (32.2) |  |
| ≥2 | 186 (33.2) | 2,463 (20.8) |  |
| CCI score | 2.60 ± 1.86 | 1.92 ± 1.19 | <0.001 |
| bDMARDs ever user | 14 (2.5) | 700 (5.9) | 0.001 |
| Methotrexate use | 225 (40.1) | 5,058 (42.7) | 0.24 |
| Dosage of Methotrexate (mg/week) | 10.00 ± 3.23 | 9.82 ± 2.98 | 0.38 |
| dosage=0 | 336 (59.9) | 6,778 (57.4) | 0.49 |
| 0<dosage<10 | 105 (18.7) | 2,347 (19.9) |  |
| dosage≥10 | 120 (21.4) | 2,694 (22.8) |  |
| Hydroxychloroquine | 311 (55.4) | 6,705 (56.7) | 0.60 |
| Sulfasalazine | 104 (18.5) | 2,600 (22.0) | 0.06 |
| Bucillamine | 40 (7.1) | 697 (5.9) | 0.26 |
| Leflunomide | 39 (7.0) | 648 (5.5) | 0.16 |
| Tacrolimus | 3 (0.5) | 84 (0.7) | 1.00 |
| Oral corticosteroids use | 404 (72.0) | 8,447 (71.4) | 0.78 |
| Dosage of corticosteroid (mg/day) | 7.35 ± 5.07 | 7.43 ± 4.77 | 0.78 |
| dosage=0 | 157 (31.7) | 3,389 (33.3) | 0.12 |
| 0<dosage<5 | 72 (14.6) | 1,172 (11.5) |  |
| dosage≥5 | 266 (53.7) | 5,608 (55.2) |  |
| NSAIDs use | 522 (93.1) | 10,844 (91.6) | 0.26 |

Data are expressed by mean ± SD or number with percentage

SD: standard deviation, CCI: Charlson comorbidity index, bDMARDs: biologic disease-modifying anti-rheumatic drugs, nbDMARDs: non-biologic disease-modifying anti-rheumatic drugs, NSAIDs: non-steroidal anti-inflammatory drugs
